# FGF23 and Vitamin D Metabolism

**DOI:** 10.1002/jbm4.10558

**Published:** 2021-10-13

**Authors:** Nejla Latic, Reinhold G. Erben

**Affiliations:** ^1^ Department of Biomedical Sciences University of Veterinary Medicine Vienna Austria

**Keywords:** 1α‐HYDROXYLASE, FIBROBLAST GROWTH FACTOR‐23, KLOTHO, PARATHYROID HORMONE, VITAMIN D, VITAMIN D METABOLISM

## Abstract

Apart from its phosphaturic action, the bone‐derived hormone fibroblast growth factor‐23 (FGF23) is also an essential regulator of vitamin D metabolism. The main target organ of FGF23 is the kidney, where FGF23 suppresses transcription of the key enzyme in vitamin D hormone (1,25(OH)_2_D) activation, 1α‐hydroxylase, and activates transcription of the key enzyme responsible for vitamin D degradation, 24‐hydroxylase, in proximal renal tubules. The circulating concentration of 1,25(OH)_2_D is a positive regulator of FGF23 secretion in bone, forming a feedback loop between kidney and bone. The importance of FGF23 as regulator of vitamin D metabolism is underscored by the fact that in the absence of FGF23 signaling, the tight control of renal 1α‐hydroxylase fails, resulting in overproduction of 1,25(OH)_2_D in mice and men. During recent years, big strides have been made toward a more complete understanding of the mechanisms underlying the FGF23‐mediated regulation of vitamin D metabolism, especially at the genomic level. However, there are still major gaps in our knowledge that need to be filled by future research. Importantly, the intracellular signaling cascades downstream of FGF receptors regulating transcription of 1α‐hydroxylase and 24‐hydroxylase in proximal renal tubules still remain unresolved. The purpose of this review is to highlight our current understanding of the molecular mechanisms underlying the regulation of vitamin D metabolism by FGF23, and to discuss the role of these mechanisms in physiology and pathophysiology. © 2021 The Authors. *JBMR Plus* published by Wiley Periodicals LLC. on behalf of American Society for Bone and Mineral Research.

## Introduction

Fibroblast growth factor‐23 (FGF23) was discovered in the year 2000 as a novel member of the fibroblast growth factor family. More or less at the same time, FGF23 was described in the murine brain in thalamic nuclei,^(^ ^1^ ^)^ while putative gain‐of‐function mutations in the *FGF23* gene were independently identified as the genetic cause of autosomal dominant hypophosphatemic rickets (ADHR), an inherited renal phosphate‐wasting disease.^(^ ^2^ ^)^ The latter finding turned out to be a seminal discovery with major impact in the field of bone and mineral homeostasis.

The strong link between FGF23 and vitamin D metabolism became evident soon after the initial description of FGF23 as a putative phosphaturic hormone. Shimada and colleagues^(^ ^3^ ^)^ isolated FGF23 as pathogenetic principle from rare human tumors causing tumor‐induced osteomalacia (TIO), another renal phosphate wasting disease. Injection of recombinant FGF23 into mice revealed two major actions of this factor: one action was the induction of renal phosphate wasting, resulting in hypophosphatemia, whereas the other was a distinct suppression of renal 1α‐hydroxylase (CYP27B1) mRNA expression.^(^ ^3^ ^)^ Renal 1α‐hydroxylase is the key enzyme for vitamin D hormone (1,25(OH)_2_D) production. Later on, knockout experiments in mice revealed that *Fgf23*‐deficient mice were characterized by unleashed production of 1,25(OH)_2_D, causing hypercalcemia, hyperphosphatemia, ectopic calcifications, and early lethality.^(^ ^4^, ^5^ ^)^ Similarly, loss‐of‐function mutations in *FGF23* in humans cause tumoral calcinosis, a disease associated with elevated circulating 1,25(OH)_2_D levels, hypercalcemia, hyperphosphatemia, and progressive soft tissue calcifications from which the name of the disease was coined.^(^ ^6^, ^7^ ^)^ Hence, in the absence of FGF23 the tight endocrine control of renal 1α‐hydroxylase transcription completely fails, leading to uncontrolled production of 1,25(OH)_2_D in mice and men. These findings have firmly established the essential role of FGF23 in vitamin D metabolism.

The purpose of this review to highlight our current understanding of the molecular mechanisms underlying the regulation of vitamin D metabolism by FGF23, and to discuss the role of these mechanisms in physiology and pathophysiology.

## Vitamin D Metabolism—A Brief Overview

Vitamin D is an essential molecule for bone and mineral homeostasis in most vertebrates.^(^ ^8^ ^)^ Vitamin D exists in two forms: cholecalciferol or vitamin D_3_ is derived from cholesterol in animals, whereas ergocalciferol or vitamin D_2_ is derived from ergosterol in fungi and protozoa. Both forms are referred to as vitamin D in this review. Depending on the species and the environmental conditions, vitamin D is either produced by a UVB‐mediated conversion of 7‐dehydrocholesterol in the skin, or it is taken up by ingestion of nutrients rich in vitamin D. Cutaneously synthesized or orally ingested vitamin D is then enzymatically converted to its most abundant circulating form, 25‐hydroxyvitamin D (25OHD), in the liver. This process is mediated by the high‐affinity cytochrome enzyme CYP2R1. 25(OH)D is released into the plasma and circulates in the blood mainly bound to the vitamin D–binding protein (DBP). However, in order for vitamin D to exert its biological effects, a subsequent hydroxylation step is necessary, which occurs in the kidney, the major source of circulating 1,25(OH)_2_D under physiological conditions.^(^ ^9^ ^)^ In renal proximal tubular epithelium, 1α‐hydroxylase converts 25(OH)D to its active form 1,25(OH)_2_D.^(^ ^10^ ^)^ Because 1,25(OH)_2_D is a lipophilic molecule it is able to cross cell membranes and to bind to its specific receptor, the vitamin D receptor (VDR), located in the cytoplasm and/or the nucleus of target cells. Ligand‐bound VDR forms a heterodimer with the retinoid X receptor (RXR). This complex can then bind to VDREs (vitamin D–responsive elements) to regulate gene transcription. The VDR is expressed in a wide range of tissues. Most mammalian cell types cells respond to 1,25(OH)_2_D exposure, with about 3% of the human genome regulated, directly or indirectly, by the vitamin D endocrine system.^(^ ^11^ ^)^ It is currently believed that all actions of the vitamin D endocrine system are mediated through the VDR.^(^ ^12^ ^)^

**Fig. 1:**
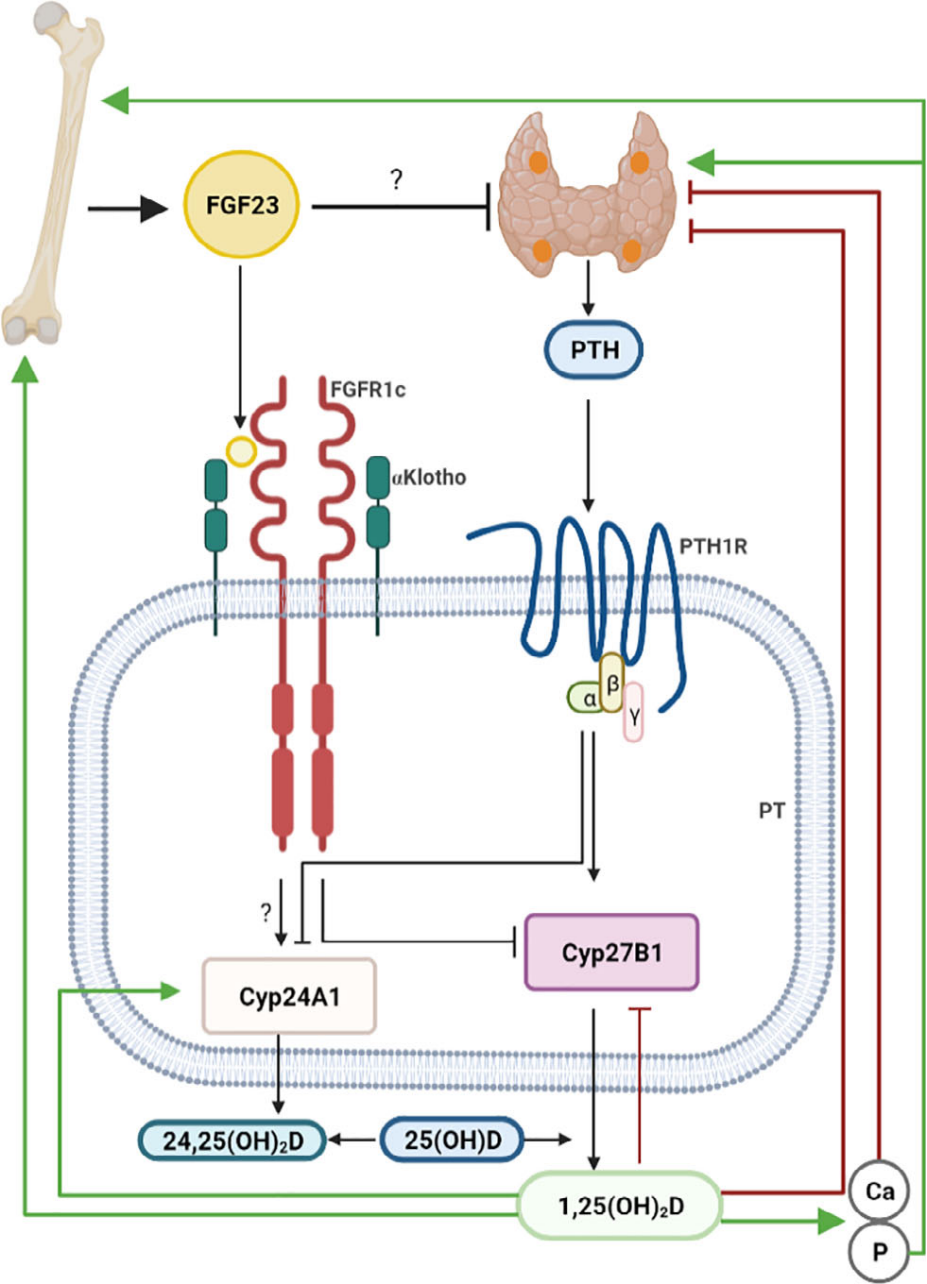
Schematic representation of FGF23 and vitamin D metabolism in the kidney. FGF23 is mainly produced in bone. Secretion of FGF23 is stimulated by the vitamin D hormone (1,25(OH)_2_D) and phosphate (P). FGF23 binds to FGFR1c in the presence of the co‐receptor αKlotho in PTs. FGF23 inhibits the expression of 1α‐hydroxylase *(Cyp27B1*), the key enzyme for vitamin D production while stimulating the catabolic enzyme 24‐hydroxylase (*Cyp24A1*). 25‐hydroxyvitamin D (25(OH)D) originating from the bloodstream can be converted into 1,25(OH)_2_D by 1α‐hydroxylase or into 24,25(OH)_2_D by 24‐hydroxylase. 1,25(OH)_2_D increases calcium (Ca) and P concentrations in the blood by stimulating their intestinal absorption. PTH is secreted in response to low plasma calcium or an increase in extracellular phosphate. Whether FGF23 is able to directly inhibit PTH production is still controversial. PTH binds to PTH1R in the kidney and promotes 1,25(OH)_2_D synthesis by upregulating *Cyp27B1* and suppressing *Cyp24A1* expression. 1,25(OH)_2_D regulates its own production by stimulating 24‐hydroxylase and inhibiting 1α‐hydroxylase activity. Arrows indicate the direction and nature of regulation. Dark red and green arrows represent direct actions of 1,25(OH)_2_D. Created with BioRender.com. FGF23 = fibroblast growth factor 23; FGFR1c = FGF receptor‐1c; PT = proximal renal tubule; PTH = parathyroid hormone; PTH1R = parathyroid hormone‐1 receptor.

The major physiological function of 1,25(OH)_2_D is the stimulation of intestinal absorption of calcium and phosphate. Overproduction of 1,25(OH)_2_D can lead to life‐threatening hypercalcemia and hyperphosphatemia. Therefore, synthesis and degradation of 1,25(OH)_2_D are tightly regulated. The major regulation of 1,25(OH)_2_D synthesis takes place at the level of 1α‐hydroxylation in the kidney, which is the rate‐limiting step in vitamin D hormone activation. In contrast, there is only a little regulation of 25‐hydroxylation in the liver. The major endocrine regulators of vitamin D metabolism are parathyroid hormone (PTH), FGF23, and 1,25(OH)_2_D itself. PTH is secreted in response to low plasma calcium and promotes 1,25(OH)_2_D synthesis by upregulating *CYP27B1* expression in the kidney (Fig. 1). Conversely, FGF23 downregulates the expression of *CYP27B1*, thereby reducing 1,25(OH)_2_D production (Fig. 1). Additionally, in a negative feedback loop, 1,25(OH)_2_D can regulate its own synthesis by inhibiting renal *CYP27B1*. An important part of the homeostatic control of circulating and intracellular concentrations of 1,25(OH)_2_D is the regulation of the vitamin D inactivation pathway. Catabolism of 25(OH)D and 1,25(OH)_2_D is induced by 24‐hydroxylation (CYP24A1).^(^ ^13^ ^)^ All endocrine regulators of vitamin D metabolism regulate 1α–hydroxylase and 24‐hydroxylase expression in the kidney in a reciprocal manner.^(^ ^14^ ^)^ The stimulator of 1α–hydroxylase expression, PTH, suppresses CYP24A1 expression, whereas the suppressors of 1α–hydroxylase, FGF23 and 1,25(OH)_2_D, stimulate 24‐hydroxylase expression (Fig. 1).^(^ ^13^ ^)^ Lessons we have learned from knockout mice and from rare mutations in humans have clearly shown that the function of CYP24A1 is essential for vitamin D metabolism, because loss‐of‐function of this enzyme results in 1,25(OH)_2_D intoxication.^(^ ^15^, ^16^ ^)^ Hence, the regulation of circulating and intracellular concentrations of 1,25(OH)_2_D occurs at both the level of activation and detoxification pathways.

## FGF23 Signaling

Within the gene family of fibroblast growth factors (FGFs), FGF23 belongs to the group of endocrine FGFs, together with FGF19 and FGF21.^(^ ^17^ ^)^ FGF23 is found in all vertebrates.^(^ ^17^ ^)^ Intact FGF23 circulating in the bloodstream is a 32‐kDa glycoprotein consisting of 227 amino acids. Only the intact form of FGF23 is biologically active. The main production site of this hormone are osteoblasts and osteocytes in bone.^(^ ^18^ ^)^

Canonical, paracrine FGFs contain a heparan sulfate‐binding site, which mediates binding of these FGFs to the local extracellular matrix, and also facilitates binding of paracrine FGFs to FGF receptors at the cell surface. All FGFs signal through four different FGF receptors (FGFRs), FGFR1, FGFR2, FGFR3, and FGFR4. FGF receptors are tyrosine kinase receptors, inducing phosphorylation of downstream molecules after ligand binding.^(^ ^19^ ^)^ In contrast to paracrine FGFs, endocrine FGFs lack a functional heparan sulfate‐binding domain, and require the presence of the transmembrane co‐receptor proteins αKlotho and βKlotho for high affinity binding to FGFRs.^(^ ^20^, ^21^, ^22^, ^23^ ^)^ αKlotho was originally discovered as an anti‐aging protein.^(^ ^24^ ^)^ However, it was later found that αKlotho functions as co‐receptor for FGF23 signaling.^(^ ^20^, ^25^ ^)^ A solid amount of data suggests that FGFR1c is the main FGFR mediating FGF23 signaling under physiological conditions.^(^ ^20^, ^22^ ^)^ The b and c isoforms of FGFR1, FGFR2, and FGFR3 are generated by alternative splicing, and show major difference in ligand binding specificity.^(^ ^22^ ^)^

The kidney is the most important target organ of FGF23 under physiological conditions. In proximal renal tubules, FGF23 signaling regulates the membrane abundance of sodium‐phosphate co‐transporters.^(^ ^26^, ^27^, ^28^ ^)^ FGF23 acts directly on proximal renal tubules.^(^ ^29^, ^30^ ^)^ Binding of blood‐borne FGF23 to the basolateral αKlotho/FGFR1c complex downregulates the apical cell membrane abundance of the phosphate co‐transporters NaPi‐2a and NaPi‐2c via activation of extracellular signal‐regulated kinase 1 and 2 (ERK1/2), serum/glucocorticoid‐regulated kinase‐1 (SGK1), and subsequent phosphorylation of the scaffolding protein Na^+^/H^+^ exchange regulatory cofactor (NHERF)‐1.^(^ ^29^ ^)^ Phosphorylation of NHERF‐1 causes internalization and degradation of NaPi‐2a/c,^(^ ^31^, ^32^ ^)^ thereby reducing tubular reabsorption of phosphate. Similar to FGF23, PTH, the other major phosphaturic hormone, downregulates renal tubular phosphate reabsorption by inducing NHERF‐1 phosphorylation via PTH receptor‐mediated activation of protein kinase A (PKA) and protein kinase C (PKC).^(^ ^32^ ^)^ Besides this phosphaturic action, FGF23 also suppresses the transcription of 1α‐hydroxylase in proximal renal tubules which is described in section Regulation of Renal Proximal Tubular 1,25(OH)_2_D Synthesis by FGF23.

## Regulation of FGF23 Secretion in Bone by 1,25(OH) _2_D

FGF23 secretion in osteoblasts and osteocytes is regulated at the transcriptional and posttranscriptional level by a plethora of local and systemic factors.^(^ ^33^ ^)^ Among other factors, 1,25(OH)_2_D, phosphate, PTH, and pro‐inflammatory cytokines have been postulated to play a crucial role in the regulation of bony FGF23 secretion. However, the molecular mechanisms underlying this regulation are still controversial. In vitro treatment of osteoblastic cells isolated from normal and uremic rats with 1,25(OH)_2_D increased *Fgf23* transcriptional activity and FGF23 secretion.^(^ ^34^, ^35^ ^)^ When the cells were treated with a medium containing only phosphate the effect was no longer present, suggesting that phosphate on its own cannot stimulate FGF23 production.^(^ ^34^ ^)^ This finding has been confirmed by other investigators.^(^ ^36^, ^37^ ^)^ Kolek and colleagues^(^ ^35^ ^)^ observed that 1,25(OH)_2_D treatment of UMR‐106 osteoblast‐like cells induces a dose and time‐dependent increase in *Fgf23* mRNA levels. Simultaneous treatment with a DNA transcription inhibitor and 1,25(OH)_2_D blunted the effect of 1,25(OH)_2_D on *Fgf23* mRNA levels, suggesting that the regulation occurs at a transcriptional level.

Indeed, in mice globally lacking VDR and in mice with a specific deletion of the VDR in chondrocytes plasma levels of FGF23 were lower than in wild‐type (WT) mice.^(^ ^38^ ^)^ Moreover, these animals did not respond to treatment with 1,25(OH)_2_D, indicating that the VDR is necessary for regulation of FGF23. This was further corroborated by a recent study in mice with deletion of the *Cyp27b1* gene. FGF23 levels were low in these animals, but exogenous treatment with calcitriol increased circulating FGF23.^(^ ^39^ ^)^

It is still unclear whether 1,25(OH)_2_D directly stimulates *Fgf23* transcription or if an intermediary factor is necessary. Although Ito and colleagues^(^ ^34^ ^)^ were not able to identify VDREs in the promoter region of the mouse *Fgf23* gene, six VDREs were identified in the human *FGF23* gene that were capable of mediating direct transcriptional activation of a heterologous reporter gene by the VDR/1,25(OH)_2_D complex.^(^ ^40^ ^)^ Lee and colleagues^(^ ^41^ ^)^ deleted an enhancer region located directly upstream of the *Fgf23* promoter in mice. This regulatory region in the murine *Fgf23* promoter was identified in earlier chromatin immunoprecipitation sequencing (ChIP‐Seq) experiments.^(^ ^42^ ^)^ In mice with the proximal enhancer deletion the upregulation of *Fgf23* transcription induced by 1,25(OH)_2_D injection as well as by an acute dietary phosphate challenge was lost, showing that this enhancer region participates in the 1,25(OH)_2_D‐mediated transcriptional regulation of *Fgf23* in vivo. However, the 1,25(OH)_2_D‐induced upregulation of *Fgf23* transcription may be indirect via other transcription factors, because ChIP‐Seq analysis in an osteocyte‐like cell line failed to provide direct evidence for VDR binding to regulatory regions in the *Fgf23* locus.^(^ ^43^ ^)^

The secretion of bioactive, intact FGF23 is not only regulated at the transcriptional, but also at the posttranslational level by the balance between phosphorylation and glycosylation within or near the site of furin‐mediated cleavage.^(^ ^44^ ^)^ O‐glycosylation within the cleavage site protects FGF23 from cleavage during the secretory process, whereas phosphorylation near the cleavage site facilitates cleavage by preventing glycosylation.^(^ ^44^ ^)^ Because furin‐mediated cleavage occurs to some extent also during the normal secretion process, the balance between phosphorylation and glycosylation determines the relative amounts of intact and cleaved FGF23 secreted by osteoblasts and osteocytes. Several independent lines of evidence have suggested that extracellular phosphate is able to regulate posttranslational processing of FGF23.^(^ ^41^, ^45^, ^46^ ^)^ It is currently not known whether 1,25(OH)_2_D may not only be a transcriptional, but also a posttranslational regulator of FGF23 secretion.

## Regulation of Renal Proximal Tubular 1,25(OH)_2_D Synthesis by FGF23

As mentioned above in the Introduction, the pivotal role of the FGF23‐Klotho signaling axis for the regulation of renal 1α‐hydroxylase is illustrated by the fact that despite hypercalcemia and suppressed PTH, 1α‐hydroxylase expression levels remain high in *αKlotho*‐deficient and *Fgf23*‐deficient mice,^(^ ^4^, ^47^, ^48^ ^)^ leading to uncontrolled production of 1,25(OH)_2_D. Similarly, humans with loss‐of‐function mutations in *αKLOTHO* ^(^ ^49^ ^)^ or *FGF23* ^(^ ^7^ ^)^ are characterized by increased circulating 1,25(OH)_2_D. The sequelae of excessive 1,25(OH)_2_D synthesis are hypercalcemia, hyperphosphatemia, and extensive calcifications of soft tissues and blood vessels. The crucial pathophysiological role of excessive 1,25(OH)_2_D synthesis in diseases characterized by loss‐of‐function of FGF23/Klotho signaling is underscored by the fact that the phenotype of *αKlotho*‐deficient and *Fgf23*‐deficient mice can almost completely be rescued by concomitant ablation of vitamin D signaling.^(^ ^50^, ^51^, ^52^ ^)^

Current knowledge about the molecular mechanisms involved in the FGF23‐mediated suppression of 1α‐hydroxylase transcription in proximal tubular epithelium is still fragmentary. The almost identical phenotypes of *αKlotho* ^−/−^ and *Fgf23* ^*−/−*^ mice strongly suggest that FGF23 regulates 1α‐hydroxylase expression by an *α*Klotho‐dependent mechanism. In addition, it has been shown that a specific deletion of *Fgfr1* in proximal renal tubules blunts the FGF23‐induced suppression of 1,25(OH)_2_D production in mice.^(^ ^53^ ^)^ Hence, there is good evidence that the FGF23‐induced suppression of 1α‐hydroxylase expression involves the FGFR1c/*α*Klotho receptor complex. However, FGFR3 and FGFR4 may also play some role, because it has been shown that ablation of *Fgfr3* and *Fgfr4* reduces the FGF23‐mediated suppression of renal 1α‐hydroxylase expression in *Hyp* mice, a model of excessive endogenous skeletal secretion of FGF23.^(^ ^54^ ^)^ In addition, the suppression of serum 1,25(OH)_2_D levels by injection of recombinant FGF23 was similar in kidney‐specific conditional *Fgfr1* knockout mice compared with global *Fgfr3* ^*−/−*^ and *Fgfr4* ^*−/−*^ mutant mice.^(^ ^55^ ^)^ Proximal renal tubules express FGFR1, FGFR3, and FGFR4, but only a little FGFR2.^(^ ^29^, ^55^ ^)^ Taken together, these findings suggest that FGF23 suppresses renal 1α‐hydroxylase expression by an *α*Klotho dependent signaling mechanism involving FGFR1, FGFR3 and FGFR4, with FGFR1c probably being the most important receptor under physiological conditions.

There is good evidence that the signaling events downstream of FGFRs involve ERK1/2 activation. The elevated circulating FGF23 levels in *Hyp* mice are associated with increased renal ERK1/2 signaling, and ERK1/2 inhibition increases 1,25(OH)_2_D production in *Hyp* mice in vivo.^(^ ^56^, ^57^ ^)^ The signaling pathway further downstream of ERK1/2 is not known. Urakawa and colleagues^(^ ^20^ ^)^ established the transcription factor egr‐1 as one of the downstream targets of FGF23 signaling in the kidney. However, egr‐1 does not seem to be involved in the FGF23‐mediated regulation of renal 1α‐hydroxylase, because the FGF23‐mediated suppression of 1α‐hydroxylase mRNA expression was found to be similar in WT and *egr‐1* ^*−/−*^ mice.^(^ ^58^ ^)^ In addition, the FGF23‐mediated suppression of 1α‐hydroxylase mRNA abundance has been shown to be VDR‐independent, as evidenced by the fact that it is not blunted in VDR deficient mice.^(^ ^59^ ^)^

At the genomic level, the work of Meyer and colleagues^(^ ^60^ ^)^ has significantly advanced our understanding of the gene regulatory networks involved in the endocrine control of 1α‐hydroxylase transcription. ChIP‐Seq experiments in mice treated with recombinant FGF23 have shown that FGF23 controls the transcriptional activity of the 1α‐hydroxylase gene in the kidney through kidney‐specific regulatory elements located in introns of the neighboring *Mettl21b* gene.^(^ ^60^ ^)^ Moreover, deletion of the enhancer regions in the *Mettl21b* gene resulted in complete resistance to FGF23 treatment, providing firm evidence that FGF23 suppresses 1α‐hydroxylase gene transcription through these kidney‐specific enhancer sites.^(^ ^60^ ^)^ However, the transcription factor(s) binding to these enhancer regions, and, thus, the exact regulatory pathways are still unknown. In addition, it is important to note that mice with a deletion of all three enhancers in the *Mettl21b* gene do not recapitulate the phenotype of *Fgf23*‐deficient or *αKlotho*‐deficient mice which are characterized by a 1,25(OH)_2_D intoxication.^(^ ^60^ ^)^ Therefore, there are still gaps in our understanding of the suppressive effect of FGF23 signaling on renal 1α‐hydroxylase.

It is well established that the suppression of renal 1α‐hydroxylase induced by injection of recombinant FGF23 into WT mice is accompanied by an upregulation of 24‐hydroxylase mRNA expression in proximal renal tubules.^(^ ^28^ ^)^ However, it still awaits further clarification whether FGF23 signaling is able to directly regulate 24‐hydroxylase in the kidney in a VDR‐independent manner, or whether this regulation occurs via alterations in 1,25(OH)_2_D production and VDR signaling. Due to the fact that 1,25(OH)_2_D is a strong regulator of 24‐hydroxylase transcription, it is difficult to dissect direct and indirect effects of FGF23 signaling on 24‐hydroxylase in vivo. Experiments in VDR‐deficient mice suggested that the FGF23‐mediated regulation of 24‐hydroxylase is entirely VDR‐dependent, because treatment of these mice with recombinant FGF23 suppressed 1α‐hydroxylase, but failed to upregulate 24‐hydroxylase expression.^(^ ^59^ ^)^ In agreement with these data, the FGF23‐mediated induction of 24‐hydroxylase is completely lost in kidney‐specific 1α‐hydroxylase knockout mice unable to respond to FGF23 treatment with altered 1,25(OH)_2_D production.^(^ ^60^ ^)^ However, a potential pitfall in experiments with VDR‐deficient and 1α‐hydroxylase–deficient mice is that 24‐hydroxylase expression is profoundly suppressed in the absence of vitamin D signaling, which may mask acute effects of FGF23 on 24‐hydroxylase transcription.

Recent evidence in favor of an at least partially VDR independent effect of FGF23 signaling on 24‐hydroxylase expression came from a mouse model in which an enhancer region downstream of the 24‐hydroxylase gene was deleted. In mice with this deletion, the FGF23‐mediated induction of 24‐hydroxylase expression was blunted. In contrast, the 1,25(OH)_2_D‐mediated induction of 24‐hydroxylase remained normal, suggesting that FGF23 and 1,25(OH)_2_D may have independent effects on *Cyp24a1* expression.^(^ ^61^ ^)^ However, the exact mechanisms of the regulation of renal 24‐hydroxylase by FGF23 still remain obscure.

## Physiological and Pathophysiological Role of FGF23‐Mediated Regulation of Vitamin D Metabolism

When the regulations at the level of the individual organs are put into a larger framework, it becomes clear that the FGF23‐mediated control of vitamin D metabolism is part of a negative feedback loop between bone and kidney. 1,25(OH)_2_D produced in the kidney stimulates secretion of FGF23 in bone, which in turn downregulates 1,25(OH)_2_D production in the kidney (Fig. 1). The phenotypes of *Fgf23* and α*Klotho* knockout mice and that of humans with rare mutations in *FGF23* and α*KLOTHO* have clearly shown that the tonic suppression of renal 1α‐hydroxylase transcription mediated through FGF23 signaling is indispensable for the homeostatic control of vitamin D metabolism.^(^ ^4^, ^7^, ^48^, ^49^ ^)^ In the absence of FGF23 signaling, the stringent endocrine control mechanisms of 1,25(OH)_2_D synthesis and degradation fail, resulting in inappropriately high circulating 1,25(OH)_2_D levels. It is evident that a downregulation of the PTH‐induced stimulation of renal 1α‐hydroxylase transcription cannot compensate for the lack of FGF23 signaling in these situations, highlighting the essential role of FGF23 signaling for the physiology of vitamin D metabolism. It is still unclear whether circulating FGF23 robustly suppresses PTH secretion (Fig. 1). Although data from rodents suggest that FGF23 suppresses PTH secretion in vivo,^(^ ^62^, ^63^ ^)^ the relevance of this mechanism in humans has been questioned because often concomitant increases in FGF23 and PTH are found.^(^ ^64^, ^65^ ^)^

Why has nature equipped vertebrates with two phosphaturic hormones with antagonistic effects on 1α‐hydroxylase expression? Interestingly, there is good evidence from experimental and clinical studies that FGF23 and PTH interact in proximal renal tubules with regard to their phosphaturic actions in an additive or even synergistic manner.^(^ ^66^, ^67^, ^68^ ^)^ However, they profoundly differ in their effect on renal 1α‐hydroxylase expression. Teleological interpretations are always difficult to make and are often prone to error, but the principal physiological function of FGF23 may be to prevent hyperphosphatemia by increasing renal phosphate excretion and by indirectly reducing intestinal phosphate absorption through suppression of 1,25(OH)_2_D production, whereas that of PTH may be to counteract hypocalcemia. The prevention of chronic hyperphosphatemia is important for a healthy life, because this condition can lead to irreversible soft tissue calcifications with potentially serious side effects. PTH secretion is also stimulated by an increase in extracellular phosphate, but the stimulation of 1,25(OH)_2_D synthesis associated with a rise in PTH limits the capacity of PTH to deal with hyperphosphatemia. Therefore, the suppression of renal 1,25(OH)_2_D production makes FGF23 a more effective hormone for counteracting hyperphosphatemia compared with PTH. In situations with elevated PTH and subsequently increased 1,25(OH)_2_D to cope with hypocalcemia, FGF23 may fine‐tune the balance between bone resorption and intestinal absorption of calcium and phosphate by putting a brake on PTH‐stimulated 1,25(OH)_2_D production, possibly modulated by extracellular phosphate concentrations. Hence, from a systems biology point of view, it makes perfect sense to have two phosphaturic hormones with antagonistic effects on 1α‐hydroxylase expression in order to be able to cope with diverse disturbances in calcium and phosphate homeostasis.

These useful physiological homeostatic mechanisms may turn into maladaptive processes in pathophysiological situations with chronically elevated FGF23. In renal phosphate‐wasting diseases characterized by inappropriately high circulating intact FGF23 such as X‐linked hypophosphatemia (XLH), autosomal dominant hypophosphatemic rickets (ADHR), or tumor‐induced osteomalacia (TIO), the FGF23‐mediated suppression of 1,25(OH)_2_D synthesis aggravates the hypophosphatemia, because in addition to renal phosphate wasting, 1,25(OH)_2_D‐mediated intestinal absorption of phosphate is reduced. The major clinical problem in these patients are the skeletal manifestations of impaired bone mineralization due to hypophosphatemia. Therefore, the FGF23‐mediated impairment in vitamin D metabolism contributing to hypophosphatemia is an important part of the pathophysiology of renal phosphate‐wasting disorders.

In patients with chronic kidney disease (CKD) the blood concentrations of intact FGF23 rise stage‐dependently, and can reach very high levels in end‐stage renal disease.^(^ ^69^, ^70^ ^)^ The reason for the elevated bony FGF23 secretion in CKD patients is still not entirely clear. It was previously thought that the rise in circulating FGF23 is driven by hyperphosphatemia due to the diminished capacity of the kidneys to excrete phosphate. However, clinical studies have clearly shown that hyperphosphatemia or increased PTH are not the drivers of FGF23 secretion in early stage CKD patients.^(^ ^71^ ^)^ Circulating intact FGF23 is a strong and independent risk factor for CKD progression, left ventricular hypertrophy, and mortality in CKD patients,^(^ ^72^, ^73^, ^74^ ^)^ and may directly promote left ventricular hypertrophy.^(^ ^73^, ^75^ ^)^ It is clear that elevated FGF23 levels in CKD patients contribute to the suppression of 1,25(OH)_2_D production, which is already diminished due to the reduction in functional kidney mass. Whether this is good or bad is a matter of current controversy. Good aspects may be that low circulating 1,25(OH)_2_D levels help reduce intestinal phosphate absorption and do not further promote bony FGF23 secretion. However, bad aspects may be that low 1,25(OH)_2_D levels may compromise the immune system and may fail to suppress the elevated PTH secretion. This controversy has been fueled by a recent study showing that treatment of maintenance hemodialysis patients with secondary hyperparathyroidism and left ventricular hypertrophy with a vitamin D analog increased circulating intact FGF23 and aggravated left ventricular hypertrophy relative to a group treated with a calcimimetic that lowered circulating FGF23.^(^ ^76^ ^)^ These findings support the notion that treatment of CKD patients with vitamin D analogs at the expense of increased FGF23 secretion may have untoward effects on cardiovascular endpoints.

## Conclusion

The purpose of this review was to highlight the current knowledge about the molecular mechanisms underlying the regulation of vitamin D metabolism by FGF23 in health and disease. FGF23 is an essential regulator of vitamin D metabolism, suppressing 1,25(OH)_2_D production in proximal renal tubules by downregulating its synthesis through suppression of 1α‐hydroxylase expression, and activating degradation by increasing 24‐hydroxylase transcription. During recent years, major advances have been made toward a more complete understanding of the genomic mechanisms involved in the FGF23‐mediated regulation of vitamin D metabolism. However, there are still major gaps in our knowledge, as evidenced by the fact that the intracellular signaling cascades downstream of FGF receptors regulating transcription of 1α‐hydroxylase and 24‐hydroxylase in proximal renal tubules still remain undefined.

## Conflict of Interest

NL and RGE have nothing to dislose.

### Peer Review

The peer review history for this article is available at https://publons.com/publon/10.1002/jbm4.10558.
